# Discovery of Trametinib as an orchestrator for cytoskeletal vimentin remodeling

**DOI:** 10.1093/jmcb/mjae009

**Published:** 2024-03-01

**Authors:** Shuangshuang Zhao, Zhifang Li, Qian Zhang, Yue Zhang, Jiali Zhang, Gaofeng Fan, Xiaobao Cao, Yaming Jiu

**Affiliations:** Unit of Cell Biology and Imaging Study of Pathogen Host Interaction, The Center for Microbes, Development and Health, Key Laboratory of Molecular Virology and Immunology, Shanghai Institute of Immunity and Infection, Chinese Academy of Sciences, Shanghai 200031, China; Guangzhou National Laboratory, Guangzhou 510005, China; Unit of Cell Biology and Imaging Study of Pathogen Host Interaction, The Center for Microbes, Development and Health, Key Laboratory of Molecular Virology and Immunology, Shanghai Institute of Immunity and Infection, Chinese Academy of Sciences, Shanghai 200031, China; Unit of Cell Biology and Imaging Study of Pathogen Host Interaction, The Center for Microbes, Development and Health, Key Laboratory of Molecular Virology and Immunology, Shanghai Institute of Immunity and Infection, Chinese Academy of Sciences, Shanghai 200031, China; University of Chinese Academy of Sciences, Beijing 100049, China; School of Life Science and Technology, ShanghaiTech University, Shanghai 201210, China; School of Life Science and Technology, ShanghaiTech University, Shanghai 201210, China; Guangzhou National Laboratory, Guangzhou 510005, China; Unit of Cell Biology and Imaging Study of Pathogen Host Interaction, The Center for Microbes, Development and Health, Key Laboratory of Molecular Virology and Immunology, Shanghai Institute of Immunity and Infection, Chinese Academy of Sciences, Shanghai 200031, China; School of Life Science and Technology, ShanghaiTech University, Shanghai 201210, China

**Keywords:** vimentin, intermediate filaments, cytoskeleton, small molecule, network reorganization

## Abstract

The dynamic remodeling of the cytoskeletal network of vimentin intermediate filaments supports various cellular functions, including cell morphology, elasticity, migration, organelle localization, and resistance against mechanical or pathological stress. Currently available chemicals targeting vimentin predominantly induce network reorganization and shrinkage around the nucleus. Effective tools for long-term manipulation of vimentin network dispersion in living cells are still lacking, limiting in-depth studies on vimentin function and potential therapeutic applications. Here, we verified that a commercially available small molecule, trametinib, is capable of inducing spatial spreading of the cellular vimentin network without affecting its transcriptional or Translational regulation. Further evidence confirmed its low cytotoxicity and similar effects on different cell types. Importantly, Trametinib has no impact on the other two cytoskeletal systems, actin filaments and the microtubule network. Moreover, Trametinib regulates vimentin network dispersion rapidly and efficiently, with effects persisting for up to 48 h after drug withdrawal. We also ruled out the possibility that Trametinib directly affects the phosphorylation level of vimentin. In summary, we identified an unprecedented regulator Trametinib, which is capable of spreading the vimentin network toward the cell periphery, and thus complemented the existing repertoire of vimentin remodeling drugs in the field of cytoskeletal research.

## Introduction

Small-molecule inhibitors play pivotal roles as therapeutic drugs and research tools, and their identification for targeting protein activities or remodeling is a significant focus of pharmacological and biochemical studies. Cytoskeletal networks serve as multifunctional molecular machines. A considerable number of small molecules have been recognized and utilized to inhibit the dynamic assembly/disassembly of cytoskeletal proteins, e.g. Cytochalasin D used for F-actin (Osumi-Yamashita  et al., 1992; Mortensen and Larsson, 2003), Paclitaxel and Nocodazole for microtubules (Müller-Deku et al., 2020; Marquis et al., 2021), CK666 for Arp2/3 (Chánez-Paredes et al., 2019; Fokin et al., 2022), and Blebbistatin for myosin II (Roman et al., 2018; Meller et al., 2023). Therefore, small-molecule inhibitors serve as powerful tools for dissecting the dynamic regulation of cytoskeletal networks both *in vitro* and *in vivo*.

The dynamic remodeling of vimentin intermediate filaments is of great significance, providing cells with the flexibility to resist mechanical or pathological stress. The remodeling of vimentin filaments is regulated by various chemicals, such as Withaferin A (WFA) (Grin et al., 2012; Menko et al., 2014; Surolia et al., 2019), Simvastatin (Trogden et al., 2018; Lavenus et al., 2020), Ajoene (Kaschula et al., 2019), and β,β′-iminodipropionitrile (IDPN) (Schmidt et al., 2015). In general, the vimentin network radiates from the perinuclear region toward the cell periphery, interweaving into a web throughout the cytoplasm (Biskou et al., 2019). All these chemicals have been shown to facilitate the shrinkage or disruption of filamentous vimentin network around the nucleus, each with its preferred dynamics (Ramos et al., 2020). However, chemicals that could promote dispersion of the vimentin network within the cytoplasm have not been reported. This limitation hinders the study of cytoskeletal vimentin remodeling in terms of both physiological and pathological functions.

Recently, we discovered several potential chemicals that induce vimentin dispersion through an imaging-based chemical screen, among which Trametinib, a commercially available small molecule targeting mitogen-activated extracellular signal-regulated kinase (MEK), induced the most significant expansion of the vimentin network (Zhao et al., 2023). In this study, we characterized the ability of Trametinib to control the spatial dispersion of the vimentin network, in particular demonstrating its dose dependence, low cytotoxicity, and irreversible property in regulating vimentin remodeling. Based on these findings, we propose that Trametinib can be widely adopted as a laboratory chemical for manipulating the vimentin network in future cytoskeletal research.

## Results

### Trametinib leads to dispersive reorganization of the cytoskeletal vimentin network

**Figure 1:**
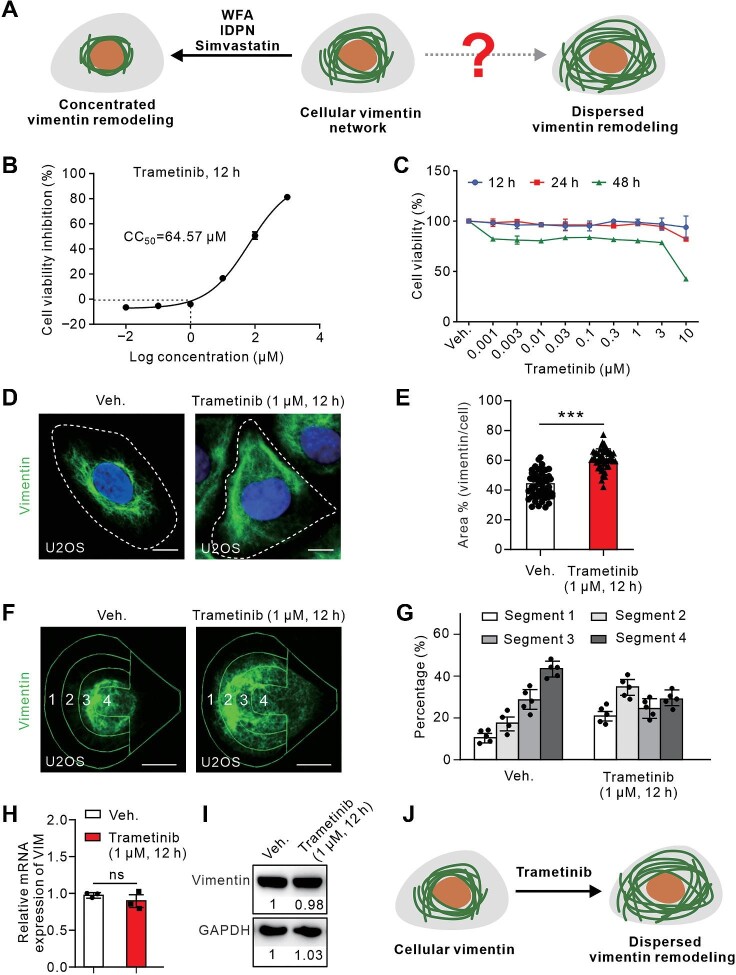
Trametinib treatment leads to dispersion of the cellular vimentin network. (**A**) Schematic diagram of vimentin remodeling. The arrowhead toward the left indicates known chemicals leading to vimentin network concentration. The arrowhead toward the right indicates unidentified tools leading to vimentin network dispersion. (**B**) The cytotoxicity assay of U2OS cells treated with Trametinib at a series of dilutions for 12 h reveals the CC_50_. (**C**) The cytotoxicity assay of U2OS cells treated with Trametinib at a series of dilutions for various durations. (**D**) Representative immunofluorescence images of U2OS cells treated as indicated and stained with vimentin antibody. White dashed lines indicate the outlines of the cell. Scale bar, 10 μm. (**E**) Quantification of the relative vimentin area to the cell area. *n = *20 views from three independent experiments. (**F**) Localization of the vimentin network in U2OS cells grown on ‘crossbow’-shaped micropatterns, visualized by vimentin antibody. Scale bar, 10 μm. (**G**) Quantification of the relative vimentin intensity within the corresponding segments. *n = *25 cells from three independent experiments. (**H**) Relative mRNA levels of vimentin. The data are from three independent experiments. (**I**) Western blot analysis of vimentin protein levels. The numbers indicate the intensity of the corresponding band. The blot was also probed with GADPH antibody to verify equal sample loading. (**J**) Schematic diagram showing that the vimentin network becomes dispersive upon Trametinib treatment. Data are presented as mean ± SD in all quantification panels. ns, no significant difference; ***P < 0.001 (unpaired two-tailed Student's *t*-test).

A number of chemicals, such as WFA (Bargagna-Mohan et al., 2015; Mohan and Bargagna-Mohan, 2016; Zhao et al., 2023), IDPN (Schmidt et al., 2015), and Simvastatin (Trogden et al., 2018; Lavenus et al., 2020), have been shown to remodel the vimentin network by concentrating the irradiated cytoplasmic vimentin filaments, but the chemicals for spreading the vimentin network are still lacking (Figure 1A).

Trametinib (GSK1120212) is an oral drug that selectively targets MEK1/2 and has been approved by US Food and Drug Administration for the treatment of metastatic melanoma (Dummer et al., 2020). Our previous work revealed that Trametinib induces dispersion of the vimentin network (Zhao et al., 2023), which prompted us to delve deeper into the effects of this drug on cytoskeletal vimentin remodeling. First, a cytotoxicity assay was conducted after treatment of Trametinib at various concentrations for 12 h, and a relatively high cytotoxic concentration 50% (CC_50_) (64.57 μM in U2OS cells) that meets the standard requirement for a laboratory chemical was identified (Figure 1B). Then, cell viability was measured after treatment of Trametinib at a series of dilutions for 12, 24, and 48 h, respectively. It was evident that Trametinib treatment for 48 h induced mild stress on cells across all doses, whereas up to 10 μM Trametinib treatment for 12 or 24 h did not show obvious toxicity (Figure 1C). These findings indicate the potential of Trametinib as a laboratory tool for manipulating the vimentin network while maintaining relative health of the treated cells.

Next, immunofluorescence was performed in U2OS cells treated with 1 μM Trametinib for 12 h, the same conditions used in the previous screen (Zhao et al., 2023). As illustrated in Figure 1D, the endogenous vimentin network was significantly expanded after Trametinib treatment, which was confirmed by quantification of the vimentin area relative to the cell area (Figure 1E). To reduce the variability in cell morphology that may interfere with the quantification of subcellular localization of the vimentin network, cells were cultured on fibronectin-coated crossbow-shaped micropatterns, where they exhibit regular morphology and vimentin organization (Vignaud et al., 2012). By dividing the cells into four segments, from segment 1 at the leading edge to segment 4 at the trailing end (Figure 1F), and quantifying the intensity of vimentin fluorescence at each cell segment, we provided quantitative validation that in vehicle-treated cells, vimentin filaments indeed accumulated at the cell center around the nucleus, while in Trametinib-treated cells, the vimentin network extended toward the leading edge of the cell (Figure 1G).

Furthermore, there were no sustained changes in either the transcriptional level or the protein quantity of endogenous vimentin, as determined by quantitative polymerase chain reaction (qPCR) and western blotting, respectively (Figure 1H and I), suggesting that the redistribution of the vimentin network following Trametinib treatment might be due to the reorganization of existing proteins rather than affecting *de novo* synthesis. In conclusion, we identified a chemical that induces significant dispersion of the cellular vimentin network with low cytotoxicity (Figure 1J).

### Orchestration of dispersed vimentin remodeling by Trametinib is cell type-unspecific

**Figure 2:**
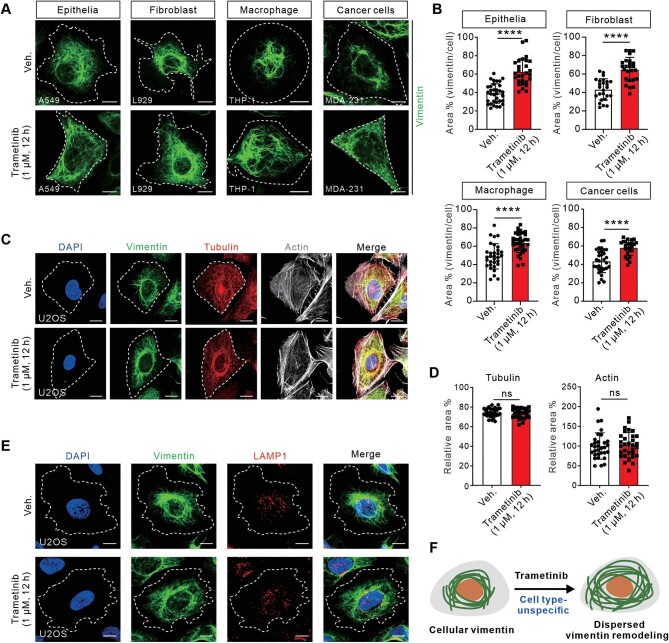
Trametinib induces vimentin network dispersion in various cell types without affecting actin and tubulin. (**A**) Representative immunofluorescence images of epithelial A549, fibroblast L929, macrophage THP-1, and breast cancer MDA-231 cells treated as indicated and stained with vimentin antibody. White dashed lines indicate the outlines of the cell. Scale bar, 10 μm. (**B**) Quantification of the relative vimentin area to the cell area in A549, L929, THP-1, and MDA-231 cells, respectively. *n = *20 views from three independent experiments. (**C**) Representative images of vimentin, microtubules, and actin filaments in U2OS cells, visualized by vimentin antibody, tubulin antibody, and phalloidin, respectively. Scale bar, 10 μm. (**D**) Quantification of the relative tubulin or actin area to the cell area in U2OS cells. *n = *20 views from three independent experiments. (**E**) Representative images of vimentin and late endosomes in U2OS cells, visualized by vimentin antibody and LAMP1 antibody, respectively. Scale bar, 10 μm. (**F**) Schematic diagram showing that vimentin dispersion upon Trametinib treatment is cell type-unspecific. Data are presented as mean ± SD in all quantification panels. ns, no significant difference; ****P < 0.0001 (unpaired two-tailed Student's *t*-test).

We then examined the effects of Trametinib on the highly ordered subcellular organization of vimentin in various cell types, including A549 lung epithelial cells, L929 mouse fibroblasts, THP-1 macrophages, and MDA-231 breast cancer cells. Representative immunofluorescence images demonstrated the dispersed vimentin network in the cells treated with Trametinib (Figure 2A), confirming a close association between vimentin dispersion and Trametinib treatment. Notably, the ratios of the vimentin area relative to the cell area were comparable across all cell types, ranging from 40%–47% to 60%–67% (Figure 2B), substantiating that Trametinib orchestrates dispersed vimentin remodeling irrespective of cell type.

Meanwhile, the actin filaments and microtubules in U2OS cells were not significantly affected by Trametinib treatment (Figure 2C and D). As dynein is involved in vimentin dispersion and also influence late endosomes, we further examined the effect of Trametinib on late endosomes by immunofluorescence for LAMP1. As shown in Figure 2E, LAMP1 puncta scattered throughout the cytoplasm, primarily concentrated near the cell nucleus, in both vehicle- and Trametinib-treated cells, suggesting that Trametinib did not impact the distribution of LAMP1 (Figure 2E). Thus, we concluded that Trametinib specifically targets vimentin for dispersion without cell-type specificity (Figure 2F).

### Orchestration of dispersed vimentin remodeling by Trametinib is rapid and efficient

**Figure 3:**
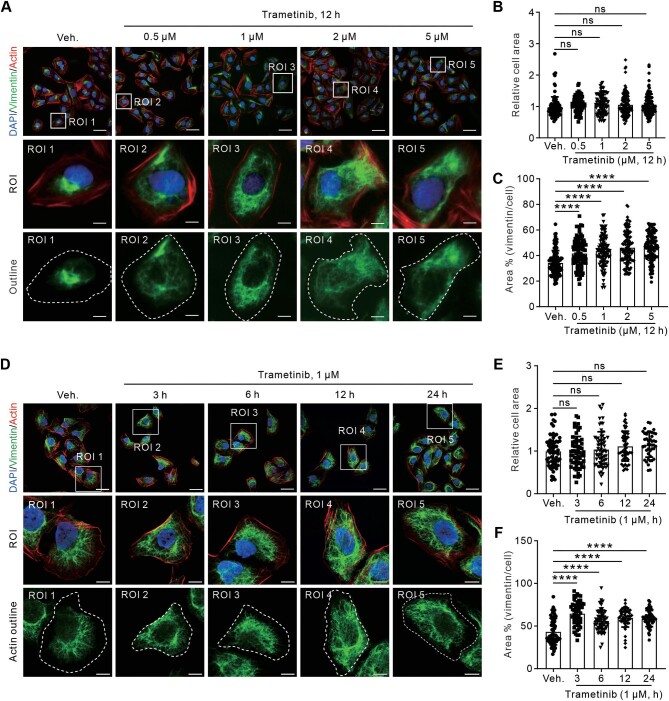
Trametinib-induced vimentin network dispersion is efficient and rapid. (**A**) Representative immunofluorescence images of U2OS cells treated with Trametinib at various concentrations for 12 h and stained with phalloidin and vimentin antibody. White dashed lines in the third row indicate the outlines of the cell. Scale bar, 10 μm. ROI, region of interest. (**B** and **C**) Quantification of the relative cell area (**B**) and the relative vimentin area to the cell area (**C**). (**D**) Representative immunofluorescence images of U2OS cells treated with 1 μM Trametinib for various durations and stained with phalloidin and vimentin antibody. White dashed lines in the third row indicate the outlines of the cell. (**E** and **F**) Quantification of the relative cell area (**E**) and the relative vimentin area to the cell area (**F**). Scale bar, 30 μm (original) and 8 μm (magnified). *n = *20 views from three independent experiments. Data are presented as mean ± SD in all quantification panels. ns, no significant difference; ****P < 0.0001 (one-way ANOVA with Dunnett's test).

To better characterize the effect of Trametinib on dispersed vimentin remodeling, U2OS cells were treated with Trametinib at various concentrations for 12 h or at 1 μM for various durations. Immunofluorescence analyses revealed that Trametinib was quite potent, inducing significant vimentin dispersion across a range of concentrations from 0.5 μM to 5 μM (Figure 3A). Quantitative results indicated that the relative cell area remained unchanged (Figure 3B), while the ratio of the vimentin area to the cell area was significantly increased after Trametinib treatment (Figure 3C). Similarly, we observed clear dispersion of vimentin after 1 μM Trametinib treatment for 3, 6, 12, and 24 h, while the relative cell area remained unaffected (Figure 3D–F). Overall, Trametinib rapidly and efficiently orchestrates dispersed vimentin remodeling.

### Orchestration of dispersed vimentin remodeling by Trametinib is relatively irreversible

**Figure 4:**
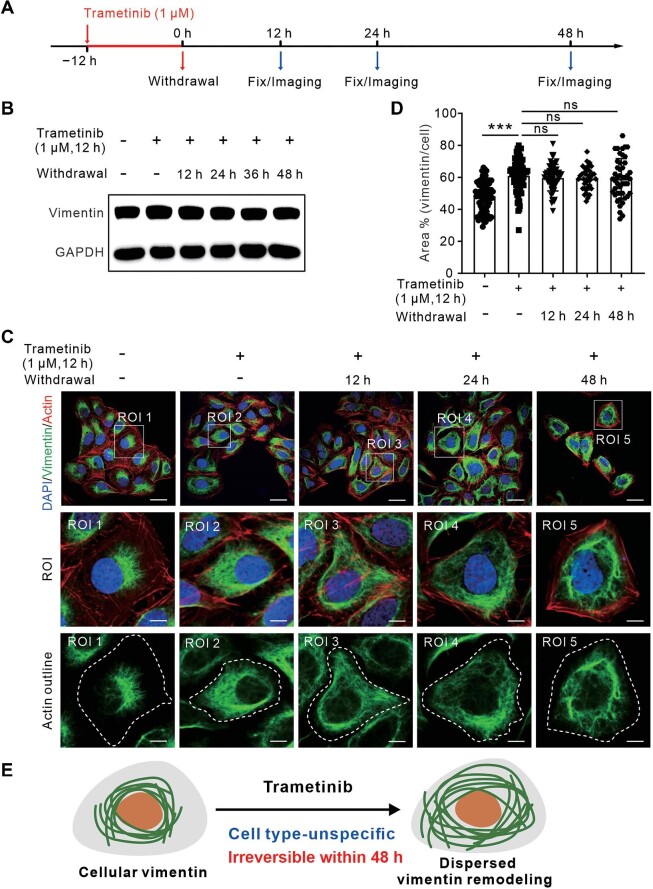
Trametinib-induced vimentin network dispersion is irreversible within 48 h. (**A**) Schematic diagram indicating the withdrawal process in U2OS cells upon 1 μM Trametinib treatment for 12 h. (**B**) Western blot analysis of vimentin protein levels. The blot was also probed with GADPH antibody to verify equal sample loading. (**C**) Immunofluorescence images of U2OS cells treated as indicated and stained with phalloidin and vimentin antibody. White dashed lines in the third row indicate the outlines of the cell. Scale bar, 30 μm (original) and 8 μm (magnified). (**D**) Quantification of the relative vimentin area to the cell area. *n = *20 views from three independent experiments. (**E**) Schematic diagram showing that vimentin dispersion upon Trametinib treatment is a cell type-unspecific, irreversible process. Data are presented as mean ± SD in all quantification panels. ns, no significant difference; ***P < 0.001 (one-way ANOVA with Dunnett's test).

To investigate the sustained effects of Trametinib on vimentin remodeling over time, we conducted a time-dependent withdrawal experiment. U2OS cells were initially treated with Trametinib at 1 μM for 12 h, after which the treatment was withdrawn by replacing with normal culture media. Vimentin organization was assessed at 12, 24, and 48 h following the withdrawal of Trametinib (Figure 4A). The total vimentin protein level remained unchanged up to 48 h after the withdrawal of Trametinib (Figure 4B). Importantly, the perinuclear accumulation of vimentin in control cells could not be recovered by withdrawing Trametinib and re-exposing the cells to normal culture media for up to 48 h (Figure 4C and D). These findings indicate that the vimentin rearrangements induced by Trametinib treatment are irreversible within 48 h (Figure 4E).

### MEK, the target of Trametinib, does not directly phosphorylate vimentin

**Figure 5:**
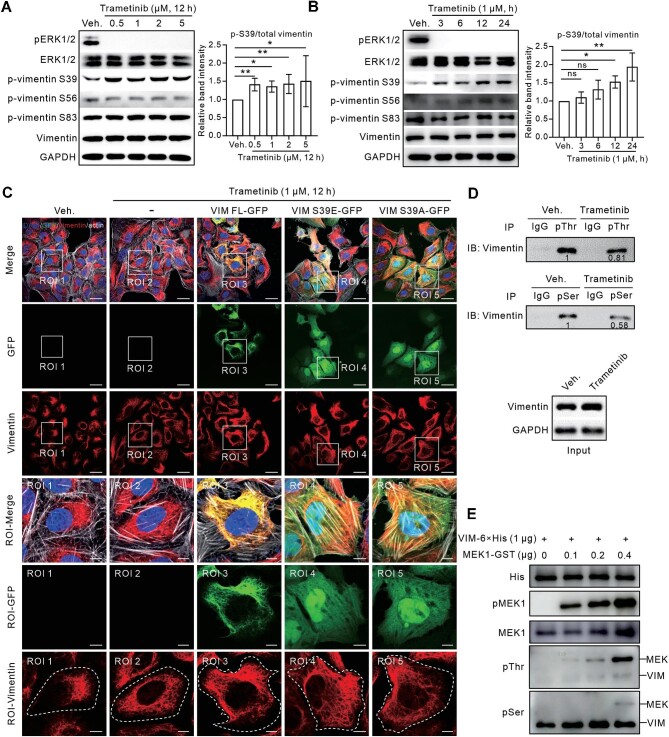
Trametinib indirectly induces vimentin phosphorylation. (**A** and **B**) Western blot analysis of phosphorylation levels of vimentin at Ser39, Ser56, and Ser83 in U2OS cells treated with Trametinib at various concentrations for 12 h (**A**) or 1 μM Trametinib for various durations (**B**). GAPDH served as the loading control. Quantification of Ser39 phosphorylation levels is shown in the right panel. (**C**) Immunofluorescence images of U2OS cells transfected with the indicated constructs and treated with 1 μM Trametinib for 12 h. White dashed lines in the last row indicate the outline of the cell. Scale bar, 30 μm (original) and 8 μm (magnified). (**D**) Immunoprecipitation (IP) assays with pThr or pSer antibody, followed by immunoblotting (IB) with vimentin antibody, were performed to detect phosphorylation levels of vimentin at threonine or serine sites, respectively, upon Trametinib treatment. (**E**) *In vitro* kinase assays with purified recombinant proteins were performed to examine phosphorylating effect of MEK1 on vimentin. Data are presented as mean ± SD in all quantification panels. ns, no significant difference; *P < 0.05, **P < 0.01 (one-way ANOVA with Dunnett's test).

It was reported that phosphorylation sites on vimentin regulate its network organization (Izawa and Inagaki, 2006). We thus examined the phosphorylation levels of vimentin at the known sites, Ser39, Ser56, and Ser83, upon Trametinib treatment. Interestingly, while the total vimentin protein level remained unaffected, the phosphorylation level at Ser39, but not at Ser56 or Ser83, was increased following Trametinib treatment (Figure 5A and B). Next, we expressed the full-length (VIM FL), phosphor-mimic (VIM S39E), or phosphor-deficient (VIM S39A) vimentin construct with a green fluorescent protein (GFP) fusion in U2OS cells. Unlike the filamentous network of VIM FL-GFP, either concentrated or dispersive, both VIM S39E-GFP and VIM S39A-GFP exhibited diffuse expression throughout the whole cytoplasm without obvious filamentous network organization (Figure 5C), indicating that the Ser39 site is critical for vimentin network organization, irrespective of whether it is phosphorylated or not. Furthermore, when VIM S39E and VIM S39A were expressed in the vimentin-knockout U2OS cells, they exhibited the similar cytoplasmic diffusion pattern as in wild-type U2OS cells, which was not changed after Trametinib treatment (data not shown). Taken together, these findings suggest that Ser39 phosphorylation might be a consequence rather than the cause of Trametinib-triggered vimentin dispersion.

Phosphorylation is one of the most important post-translational modifications (PTMs) of vimentin for manipulating its remodeling (Snider and Omary, 2014; Ostrowska-Podhorodecka et al., 2022). A total of 23 phosphorylation sites on vimentin have been identified (Shi et al., 2016). To investigate whether pan-phosphorylation of vimentin is involved, we performed immunoprecipitation assays with antibodies against phosphothreonine (pThr) or phosphoserine (pSer) to precipitate proteins pan-phosphorylated at threonine or serine sites, respectively, followed by immunoblotting for vimentin. Both pThr and pSer levels of vimentin were mildly decreased upon Trametinib treatment (Figure 5D). We further performed *in vitro* kinase assays with purified recombinant MEK and vimentin proteins but did not observe any obvious vimentin phosphorylation by MEK, the target of Trametinib (Figure 5E), suggesting that there might be intermediate regulators between Trametinib and vimentin that lead to vimentin phosphorylation.

## Discussion

While enzyme proteins with active sites are frequently targeted, proteins lacking catalytic sites can also be targeted using small molecules that disrupt their dynamics or subcellular organization. In this study, we demonstrated that Trametinib, a MEK1/2 signaling cascade inhibitor that is clinically employed to treat carcinoma (Gandara et al., 2017; Chesnokov et al., 2021; Jiang et al., 2022), holds great potential as a laboratory chemical for remodeling cytoskeletal vimentin network in living cells (Figure 4E). This discovery is particularly valuable, given the current scarcity of commonly used compounds that induce vimentin dispersion. In addition, Trametinib is commercially available at a reasonable price compared to other chemicals that promote vimentin aggregation, such as WFA and Simvastatin.

Osmotic shock is a universal stressor in multicellular organisms, especially on epithelial surfaces or during tumor metastasis. To withstand osmotic shock, organisms establish an internal aqueous environment and regulate intravascular water and electrolytes through sensitive and dynamic homeostatic mechanisms. We previously revealed that hypo-osmotic shock leads to vimentin dispersion, serving to protect the cell from collapsing under such dramatic stress (Shi et al., 2020). Here, we further confirmed that the microtubule network and dynein motor are involved in guiding vimentin dispersion during this process. Hypo-osmotic conditions were used as a positive control, compared to which Trametinib led to slower response dynamics, with the exact mechanism remains unveiled. Further investigations are necessary to unveil the potential intermediate regulators, such as other cytoskeletal components or motor proteins, between Trametinib and vimentin and understand their roles in vimentin regulation by Trametinib.

Trametinib can also be effective in a pathogenic context. For instance, the invasion of many viruses has been associated with the aggregation of vimentin in host cells (Lei et al., 2013; Zhang et al., 2020, 2022; Zheng et al., 2021; Yu et al., 2022). Our preliminary data suggest that Trametinib can disperse vimentin during the infection process, indicating a general regulatory effect of Trametinib on vimentin under both normal and pathologically stressed conditions.

PTMs play a critical role in modulating the cytoskeleton. For example, phosphorylation, acetylation, ubiquitylation, and SUMOylation have been reported to regulate actin and tubulin cytoskeletal architectures (Impens et al., 2014; Hendriks and Vertegaal, 2016). While it is evident that Trametinib modifies vimentin phosphorylation in cells, vimentin phosphorylation does not occur in the *in vitro* kinase assay using recombinant proteins, indicating the absence of direct vimentin phosphorylation by MEK. In addition, whether and how Trametinib affects other PTMs on vimentin remain elusive. Further investigations are therefore required to elucidate the underlying mechanism by which Trametinib orchestrates the remodeling of the vimentin network.

## Materials and methods

### Cell culture and plasmids

Human osteosarcoma (U2OS), MDA-231, A549, L929, and THP-1 cells were cultured at 37°C with 5% CO_2_ in Dulbecco's modified Eagle's medium (DMEM; Biological Industries) supplemented with 10% fetal bovine serum (Gibco) and 1% penicillin/streptomycin. VIM-GFP and actin-mCherry double-labeled stable-expressing U2OS cells, as well as VIM FL-GFP, VIM S39E-GFP, and VIM S39A-GFP, were kindly provided by Dr John Eriksson (University of Turku, Finland).

### Trametinib treatment

Cells were rinsed with phosphate-buffered saline (PBS) and then cultured in DMEM containing the corresponding working concentrations of Trametinib for the indicated periods at 37°C. A control (vehicle) group treated with dimethyl sulfoxide (DMSO) was included.

### Immunofluorescence

Immunofluorescence experiments were performed as previously described (Jiu et al., 2019). Briefly, cells were fixed with 4% paraformaldehyde in PBS for 15 min at room temperature, washed three times with 0.2% bovine serum albumin (BSA) in Dulbecco's PBS, and permeabilized with 0.1% Triton X-100 in PBS (PBS-T) for 5 min. Cells were blocked in 1× Dulbecco's PBS supplemented with 0.2% BSA. The following primary antibodies were used: vimentin rabbit monoclonal D21H3 antibody (dilution 1:100; #5741, Cell Signaling Technology); vimentin chicken polyclonal antibody (dilution 1:1000; ab24525, Abcam); tubulin mouse monoclonal 144 antibody (dilution 1:200; #4026, Sigma); and LAMP1 rabbit monoclonal antibody (dilution 1:100; #9091, Cell Signaling Technology). Both primary and secondary antibodies were applied to the cells, which were subsequently incubated at room temperature for 1 h. Alexa-conjugated phalloidin was added together with primary antibody solutions. All immunofluorescence data were obtained with an Olympus SpinSR10 Ixplore spinning disk confocal microscope with an UplanApo 60×/1.5 oil objective (Olympus) and a super-resolution 3D-SIM OMX SR microscope (Cytiva). For micropattern experiments, the cells were plated on CYTOOchips^TM^ prior to fixation as described previously (Jiu et al., 2019).

### Western blotting

Cells were washed with cold PBS and lysed in RIPA lysis buffer (P0013B, Beyotime) supplemented with protease and phosphatase inhibitors (P1045, Beyotime) at 4°C for 30 min. Cell debris was removed by centrifugation at 12000× *g* for 20 min at 4°C. Protein concentrations were determined by the Bradford assay kit (P0012, Beyotime). Equal amounts of lysates were resolved by sodium dodecyl sulfate (SDS)–polyacrylamide gel electrophoresis and transferred to polyvinylidene fluoride membranes (IPVH00010, Millipore). The membranes were blocked with 5% nonfat milk (8011939, BD) in PBS-T buffer at room temperature for 30 min and then incubated with primary antibodies overnight at 4°C. After being washed three times with PBS for 10 min each, the membranes were incubated for 1 h with an appropriate horseradish peroxidase-conjugated secondary antibody (dilution 1:5000; #7076V, Cell Signaling Technology). The following primary antibodies were used: vimentin rabbit monoclonal D21H3 antibody (dilution 1:1000; #5741, Cell Signaling Technology) and glyceraldehyde-3-phosphate dehydrogenase (GAPDH) rabbit monoclonal antibody (dilution 1:5000; #G8795, Sigma-Aldrich).

### Real-time qPCR

Total cellular RNA was extracted by using the EZ-press RNA Purification Kit (#B0004DP, EZBioscience) according to the manufacturer's protocols. Total RNA was reverse-transcribed by using the Color Reverse Transcription Kit (#A0010CGQ, EZBioscience). Real-time qPCR was carried out by using 2$ \times $ Color SYBR Green qPCR Master Mix (ROX2 plus) (#A0012-R2, EZBioscience) in a QuantStudio 1 system (Thermo). All readings were normalized to the level of GAPDH.

### Cytotoxicity assay

Cell proliferation and viability were measured by Cell Counting Kit-8 (C0040, Beyotime). Briefly, cells were seeded at the density of 2 × 10^3^ in 100 μl per well in a 96-well culture plate and then incubated with 10 μl of CCK-8 reagent at 37°C for 0.5–4 h. The absorbance at 450 nm was measured with the Synergy H1 Hybrid Multi-Mode Reader (BioTek).

### Immunoprecipitation

Cells were treated with DMSO or Trametinib. After 12 h, the cells were collected, washed with cold PBS, and then lysed with lysis buffer (50 mM Tris–HCl, pH 8.0, 150 mM NaCl, 0.5% NP40, 1 mM EDTA, and protease inhibitors). After centrifugation at 13800× *g* for 20 min at 4°C, the supernatant was collected and the protein concentration was determined by the BCA Protein Assay Kit (P0010, Beyotime). Meanwhile, anti-phosphoserine antibody (#05-1000, Millipore) or anti-phosphothreonine antibody (#MABS499, Millipore) was incubated with Protein A/G PLUS-Agarose (#sc-2003, Santa Cruz Biotechnology) for 1 h at room temperature. Then, equal amounts of supernatant and antibody-coupling agarose were mixed and incubated overnight at 4°C. The beads were washed twice with lysis buffer and eluted with 100 μl of SDS loading buffer at 95°C for 10 min for further immunoblotting analysis.

### *In vitro* kinase assay

For direct *in vitro* kinase assays, 1 μg of purified VIM-6×His protein and various concentrations of MEK1-GST were added to kinase buffer containing 50 mM HEPES, pH 7.2, 10 mM MnCl_2_, 150 mM NaCl, 1 mM Na_3_VO_4_, 5 mM dithiothreitol, 0.1% NP40, 100 μM adenosine triphosphate (ATP), and 10 μCi [γ-^32^P] ATP (Nuclear Iberica). The assays were conducted at 30°C for 30 min in the presence of glutathione-Sepharose beads coated with GST-Lck fusion proteins or His fusion proteins. The reaction was stopped by adding 10 μl of 6× reducing sample buffer and boiling for 10 min. The phosphorylation level was detected by western blotting.

### Statistical analysis

Statistical analysis was conducted using appropriate methods, including unpaired Student's *t*-test and one-way analysis of variance (ANOVA), as implemented in GraphPad Prism v9 software. The significance level is denoted by asterisks (*P < 0.05, **P < 0.01, ***P < 0.001, ****P < 0.0001). The presented data in quantification histograms are mean ± standard deviation (SD).

## Notes

**Author contributions:** S.Z. and Z.L. carried out the majority of experiments and interpretation of the data. Q.Z. carried out the time-dependent experiments. Y.Z. carried out the phosphorylation assays. J.Z. and G.F. carried out the *in vitro* kinase assays. X.C. and G.F. contributed during the revision. Y.J. conceived and supervised the project. Y.J. and S.Z. wrote the manuscript, with contributions from all the other authors. All the authors provided consent for publication of this study.
